# Analysis and prediction of research hotspots and trends in heart failure research

**DOI:** 10.2478/jtim-2023-0117

**Published:** 2024-07-27

**Authors:** Zeye Liu, Yuan Huang, Yang Yang, Wenchao Li, Wenhao Ju, Fengwen Zhang, Wenbin Ouyang, Shouzheng Wang, Cheng Wang, Xuanqi An, Ruibing Xia, Yakun Li, Xiangbin Pan

**Affiliations:** Department of Structural Heart Disease, National Center for Cardiovascular Disease, China & Fuwai Hospital, Chinese Academy of Medical Sciences & Peking Union Medical College, Beijing 100037, China; National Health Commission Key Laboratory of Cardiovascular Regeneration Medicine, Beijing 100037, China; Key Laboratory of Innovative Cardiovascular Devices, Chinese Academy of Medical Sciences, Beijing 100037, China; National Clinical Research Center for Cardiovascular Diseases, Fuwai Hospital, Chinese Academy of Medical Sciences, Beijing 100037, China; State Key Laboratory of Cardiovascular Disease, Fuwai Hospital, National Center for Cardiovascular Diseases, Pediatric Cardiac Surgery Center, Fuwai Hospital, Chinese Academy of Medical Sciences, and Peking Union Medical College, Beijing 100037, China; Zhengzhou University People’s Hospital, Henan Provincial People’s Hospital, Huazhong Fuwai Hospital, Pediatric Cardiac Surgery, Zhengzhou 450000, Henan Province, China; Department of Medicine I, University Hospital Munich, Ludwig-Maximilians-University Munich, Munich D-80539, Germany; Laboratory of Experimental Intensive Care and Anesthesiology, Academic Medical Center, Amsterdam 1105 AZ, The Netherlands

**Keywords:** artificial intelligence, basic, clinical, heart failure, population

## Abstract

**Background and objectives:**

Comprehensive data analyses in heart failure research can provide academics with information and help policymakers formulate relevant policies. We collected data from reports published between 1945 and 2021 to identify research topics, trends, and cross-domains in the heart failure disease literature.

**Methods:**

Text fragments were extracted and clustered from the titles and abstracts in 270617 publications using artificial intelligence techniques. Two algorithms were used to corroborate the results and ensure that they were reliable. Experts named themes and document clusters based on the results of these semiautomated methods. Using consistent methods, we identified and flagged 107 heart failure topics and 16 large document clusters (divided into two groups by time). The annual vocabularies of research hotspots were calculated to draw attention to niche research fields.

**Results:**

Clinical research is an expanding field, followed by basic research and population research. The most frequently raised issues were intensive care treatment for heart failure, applications of artificial intelligence technologies, cardiac assist devices, stem cells, genetics, and regional distribution and use of heart failure-related health care. Risk scoring and classification, care for patients, readmission, health economics of treatment and care, and cell regeneration and signaling pathways were among the fastest-growing themes. Drugs, signaling pathways, and biomarkers were all crucial issues for clinical and basic research in the entire population. Studies on intelligent medicine and telemedicine, interventional therapy for valvular disease, and novel coronavirus have emerged recently.

**Conclusion:**

Clinical and population research is increasingly focusing on the customization of intelligent treatments, improving the quality of patients’ life, and developing novel treatments. Basic research is increasingly focusing on regenerative medicine, translational medicine, and signaling pathways. Additionally, each research field exhibits mutual fusion characteristics. Medical demands, new technologies, and social support are all potential drivers for these changes.

## Introduction

Between 1990 and 2019, the global population increased by 44.6%, from 5.3 billion to 7.7 billion.^[1,2]^ Simultaneously, the number of cardiovascular disease deaths worldwide increased by 53.7%, from 12.1 million to 18.6 million.^[2,3]^ Heart failure, which is the outcome of various cardiovascular diseases, affects approximately 40 million people worldwide and has become a common health threat.^[4]^ Scientific research is required to address this challenge. Currently, funds and policy priorities in cardiovascular research (including heart failure) are increasingly focused on innovation, aiming to improve clinical outcomes.^[5]^ These trends are frequently influenced by expert opinions;^[6]^ however, an overview of previous research on heart failure at macro and global levels can enrich relevant evidence and aid decision-making.

The field of heart failure research is vast and it is very challenging to review and summarize all existing studies in the literature. The widely used journal classification systems are currently neither refined enough to identify or classify all relevant studies nor capable of elaborating on topics or concepts.^[5]^ As technology advances, semiautomated methods based on text analysis that can identify key topics^[7,8]^ offer an alternative solution to this issue. However, these methods may produce varying results because they do not rely on a predefined classification. Similar ideas have been reflected in the research and development of software such as “CiteSpace” and “VOSviewer.”^[7,8]^ To date, relevant studies have summarized the hotspots and analyzed the trends in cardiovascular research from 2004 to 2013 using natural language processing (NLP) technology in artificial intelligence methods and proposed several extremely valuable views.^[5]^ However, those studies did not focus on heart failure. Therefore, an update is needed that focuses on heart failure research trends over a relatively long period after 9 years of development.

Therefore, we aimed to identify the themes and evolutionary trends in published heart failure studies between 1945 and 2021, particularly in the last 10 years, to assess whether they have changed over time and explore the crossover among study clusters. Along with existing text mining, network analysis, and clustering methods, the relevant analysis tools were developed further to manage large datasets containing more than 270000 publications, which might be the largest and most comprehensive dataset in similar studies.

## Materials and methods

In this study, we used two complementary approaches, that is, latent Dirichlet allocation (LDA) and k-means clustering algorithm (k-means).^[9,10]^ The data were analyzed by detecting themes in publication collections and mapping document networks into clusters with identifiable research themes. Further details are available in attachments, model codes, and previous publications.^[5]^

### Data source

The dataset includes data on titles, abstracts, and keywords from 270617 heart failure publications (1945–2021). This dataset was obtained by browsing the term “heart failure” (based on medical subject heading [MeSH] phrases provided by PubMed) and decreasing the repetition rate. The data were obtained from the MEDLINE dataset of the Web of Science (WoS) Core Collection via a data license held by the Chinese Academy of Medical Sciences and Peking Union Medical College.

### Text pre-processing

We used all titles and abstracts from these publications to extract time information and noun phrases (pieces of text of various lengths) using an NLP framework developed with Python 3.9 software. Figure S1 illustrates the specific flow chart for the data analysis.

### Modelling analysis and prediction

First, we applied the LDA approach^[5,9]^ to the aforementioned text fragments from all titles and abstracts. We grouped these text fragments to identify themes and assign documents to those themes. The analysis filtered out generic terms that were commonly used in most documents, producing a series of highly specific text fragments and themes to improve accuracy and highlight class features. Five experts in the field of heart failure (all authors) named the topics according to the first 30 text fragments that represented the topics. They then cross-reviewed and merged similar topics. We further used probability analysis in the LDA model to count the number of documents contributing to the topic. Furthermore, we mapped word clouds based on keyword frequency by period (pre-2010, 2010–2020, and post-2020) to demonstrate hotspot vocabularies during varying periods.

In the second approach, we analyzed heart failure publications (2017–2018 and 2020–2021). For each period, we calculated the similarity and classified documents using an adapted cosine computation and hybrid document clustering algorithm based on text segments in the titles and abstracts of all publications.^[5,10]^ Five additional experts in the heart failure field (who were not involved in the LDA topic naming but are all included among the authors of this study) named the cluster based on the first 30 text fragments representing the cluster. We believe that the coronavirus pandemic, as a significant public health event in recent years, will have an impact on heart failure research. Since the outbreak started at the end of 2019, we chose two time periods spanning 2019 for our analysis. A ring diagram was further used to visualize the data. For each document cluster, we identified the most representative theme matching the themes generated by the LDA model.

To observe trends in relatively “niche” research areas, we performed inverse document frequency (IDF) calculations for keywords by year for research areas with few studies that were challenging to be included in the above analysis. ^[11]^ We obtained keywords with more extensive research each year in their field than in other years. Those few research directions rapidly changed over time but could be discovered.

### Statistical analysis

Two methods, LDA^[9]^ and KMeans^[10]^, were used to categorize the literature. The annual research hotness of keywords was evaluated using IDF^[11]^ as a criterion. Python 3.9 was employed for all operations in a local high-performance computing environment with no connection to any extra nets for up to 6 months. The naming of topics or categories will be done by five experts together, and the expert group will discuss each name, and the name will be confirmed only if all the expert group members all agree.

## Results

### Popular topics and emerging trends in heart failure research

**Figure 1:**
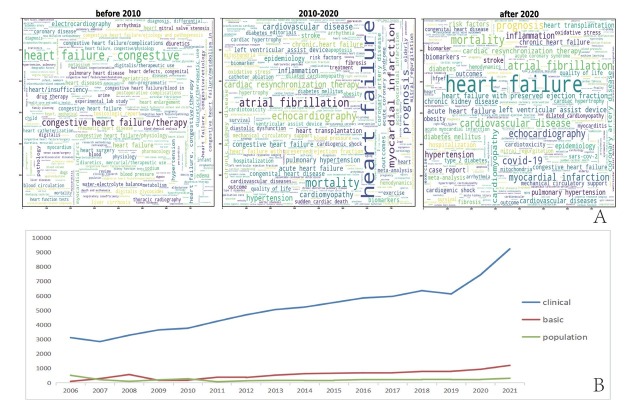
Keyword distribution with time and trends in major research areas. A. Distribution of keywords over three periods (before 2010, 2010–2020, and after 2020), where the size of the keyword reflects its frequency of appearance. B. Trends in number of studies in three categories (*i.e*., clinical, basic, and population studies) over time.

Word clouds were drawn for keywords based on the three periods (pre-2010, 2010–2020, and post-2020). The top five keywords with the highest frequency of occurrence (see the larger fonts in the figure) in each period were as follows: Pre-2010: “congestive heart failure/therapy,” “electrocardiography,” “geriatrics,” “heart failure/complications,” “hypertension;” 2010–2020: “mortality,” “congestive prognosis,” “hypertension,” “myocardial infarction,” “echocardiography;” post-2020: “mortality,” “atrial fibrillation,” “prognosis,” “COVID-19,” “echocardiography” (Figure 1A).

**Figure 2:**
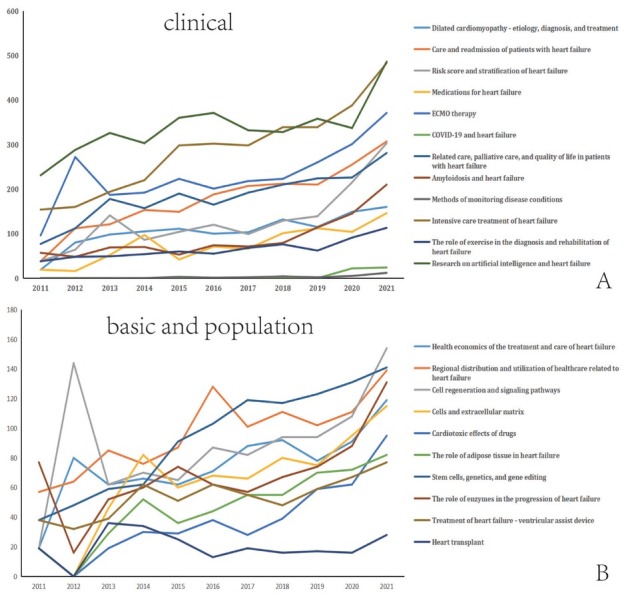
Topics with large growth (over twofold increase in number of clinical, basic, and population studies) for 2011–2021. A. For clinical research, 12 topics increased more than two-fold. B. For basic research, the 8 topics that increased more than two-fold in volume are shown; for population research only two topics had more than a two-fold increase.

We identified 107 heart failure topics, which we listed alphabetically in the Appendix (Table S1). These topics were further categorized as “clinical,” “basic,” and “demographic” studies (Figure 1B),^[5]^ such as clinical studies on the role of N-terminal (NT)-prohormone brain natriuretic peptide (BNP) in the diagnosis and treatment of heart failure, basic studies on stem cells, and population studies on health economic issues in the treatment and care of heart failure. Figure 2 presents the “hotspots” with significantly increased publications over the past 10 years. In clinical studies, intensive care treatment for patients with heart failure, applications of artificial intelligence technology, applications of extracorporeal membrane oxygenation, risk scores, and classification of heart failure were prominent, reflecting concerns for patients with end-stage heart disease and an improvement in overall medical level. These results also indicate that heart failure treatments are evolving in a more targeted and personalized direction. Among population studies, studies related to health economic issues of treatment and care and access to health-care resources are extremely “popular,” which might be due to the chronicity and continuity of heart failure treatment.^[12,13]^ Basic research in stem cells, signal transduction, enzymology, and drug toxicity were on the rise, reflecting the emergence of regenerative and translational medicine. Table 1 complements the rapidly growing themes in 2021 compared with 2011. All of the 107 LDA topics are presented in the Appendix (Table S1).

**Table 1:** Major clinical research topics in 2012 and 2021

Topic label	2012 (Number of documents)	2021 (Number of documents)
Risk score and stratification of heart failure	64	303
Treatment of the cardiorenal syndrome	304	356
Heart failure and respiratory sleep disorders	112	80
Methodology of clinical trials	224	432
Amyloidosis and heart failure	48	210
Medications for heart failure	16	146
Heart failure and serum proteins	288	519
Intensive care treatment of heart failure	160	483
Care and readmission of patients with heart failure	112	307
Heart failure and thyroid function	80	113
Psychosocial care for patients with heart failure	32	112
Extracorporeal membrane oxygenation therapy	272	371
Vasoactive drugs and the treatment of heart failure	16	103
Heart failure due to cardiomyopathy—mechanism, diagnosis, and treatment	400	640
Heart failure due to congenital heart disease	48	239
Care and patient education in heart failure	16	110
Acute coronary syndrome and myocardial injury	160	170
Heart failure after myocardial infarction—treatment and prognosis	224	407
Proteins and enzymes	32	135
Dilated cardiomyopathy—etiology, diagnosis, and treatment	80	160
Hypertension—diagnosis, monitoring, and treatment	96	201
Risk factors and predictive models for heart failure	80	146
Imaging—ultrasound, and MRI	32	249
Coagulation, anticoagulation, and atrial fibrillation	80	173
Heart failure and liver dysfunction	48	88
Treatment of acute heart failure—characteristics, influential factors, and prognosis	64	49
Coronary artery lesions and heart failure	96	185
Related care, palliative care, and quality of life in patients with heart failure	112	281
Pulmonary hypertension and heart failure	208	185
Management and rehabilitation of heart failure disease	16	89
Treatment of heart failure—diuretics	48	59
The role of exercise in the diagnosis and rehabilitation of heart failure	48	113
Heart failure and complications in infants and children	32	59
Pacing therapy for arrhythmias	64	65
Basic science and population research		
Cell regeneration and signaling pathways	144	154
Oxygen metabolism and heart failure	89	115
The role of enzymes in the progression of heart failure	16	131
Stem cells, genetics, and gene editing	48	141
Genetics and single nucleotide polymorphisms	16	61
Immune cells and immune regulation	48	72
Health economics of the treatment and care of heart failure	2012	80
Health economics of the treatment and care of heart failure	2021	119
Regional distribution and use of health care related to heart failure	2012	64
Regional distribution and use of health care related to heart failure	2021	139

### Large study areas and trends defined by document clusters

**Figure 3:**
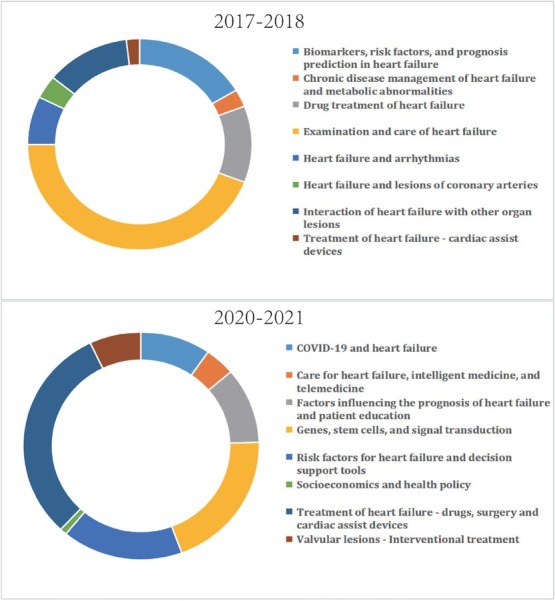
Distribution of document clusters for 2017–2018 and 2020–2021. A. In 2017–2018, the eight largest clusters accounted for 94.12% of the total publication output. B. In 2020–2021, the eight largest clusters accounted for 95.60% of the total publication output.

To corroborate our results using the LDA method, we used a complementary approach to investigate how the documents were grouped based on their textual similarity. We applied a hybrid clustering algorithm in two publication datasets (2017–2018 and 2020–2021) and obtained eight large clusters in each period, with the number of documents in the clusters comprising 94.12% and 95.60% of the total number of documents in the period, respectively. The document clusters between the two periods are compared in Figure 3. The research areas exploring risk factors for heart failure, drug treatment, cardiac assist devices, and patient management played crucial roles in both periods. Meanwhile, the emerging fields of intelligent medicine and telemedicine, interventional therapy for valvular lesions, and COVID-19 (2020–2021) demonstrate that this field is constantly evolving in response to technological advances and actual medical needs.

Table 2 compares the results obtained by the two methods, which show the high consistency of the two methods. The LDA method provided more detailed clusters for 16 document clusters. For example, the cluster “valvular lesions—interventional treatment” exhibited different areas of focus, that is, transcatheter aortic valve implantation, interventional therapy for mitral valve disease, minimally invasive surgery, and auxiliary equipment. Furthermore, the “Biomarkers, risk factors, and prognosis prediction in heart failure” cluster was also divided into NT-proBNP, serum proteins, and the renin–angiotensin–aldosterone system.

**Table 2:** Cluster names and topics withinclusters

Cluster name	Latent dirichlet allocation topics
2017–2018	
Examination and care of heart failure	Care and readmission of patients with heart failure
	Imaging—ultrasound, and MRI
	Methods of monitoring disease conditions
	Related care, palliative care, and quality of life in patients with heart failure
Heart failure and arrhythmias	Cardiac resynchronization therapy for heart failure
	Pacing therapy for arrhythmias
	Coagulation, anticoagulation, and atrial fibrillation
Biomarkers, prediction in risk heart factors, failure and prognosis	The role of NT-proBNP in the diagnosis and treatment of heart failure
	Heart failure and serum proteins
	Renin–angiotensin–aldosterone system
	Biomarkers and predictive models of heart failure
	Risk score and stratification of heart failure
Heart arteries failure and lesions of coronary	Coronary artery lesions and heart failure
	Acute coronary syndrome and myocardial injury
	Acute myocardial infarction—interventional and pharmacological treatment
Interaction of heart failure with other organ lesions	Treatment of the cardiorenal syndrome
	Heart failure and respiratory sleep disorders
	Pulmonary hypertension and heart failure
	Heart failure and thyroid function
	Heart failure and liver dysfunction
Treatment devices of heart failure—cardiac assist	Treatment of heart failure—ventricular assist device
	Extracorporeal membrane oxygenation therapy
	Minimally invasive surgery and assistive devices
Drug treatment of heart failure	Mechanism and application of antagonist drugs
	Vasoactive drugs and the treatment of heart failure
	Potassium metabolism and cardiotonic drugs—effects on patients with heart failure
	Cardiovascular effects of blood glucose control drugs
Chronic failure and disease metabolic management abnormalities of heart	Water–electrolyte balance and micronutrient supplementation in patients with heart failure
	Oxygen metabolism and heart failure
	Diabetes and heart failure
	Effect of obesity and lipids on heart failure
2020–2021	
Genes, stem cells, and signal transduction	Cell regeneration and signaling pathways
	Stem cells, genetics, and gene editing
	Cells and extracellular matrix
	Cardiomyocyte ultrastructure, energy metabolism, and signaling
	Genetics and single nucleotide polymorphisms
Socioeconomics and health policy	Health economics of the treatment and care of heart failure
	Cost and use of health-care services related to heart failure
	Public health, socioeconomics, and digital health
Factors failure and influencing patient education the prognosis of heart	Heart failure after myocardial infarction—treatment and prognosis
	Psychosocial care for patients with heart failure
	The role of exercise in the diagnosis and rehabilitation of heart failure
	Effect of ejection fraction on the treatment and prognosis of patients with heart failure
Valvular lesions—Interventional treatment	Minimally invasive treatment of heart failure—transcatheter valve replacement
	Treatment of mitral valve lesions—surgical and interventional
	Minimally invasive surgery and assistive devices
	Minimally invasive treatment for heart failure—TAVR
Treatment of heart failure—drugs, surgery, and cardiac assist devices	ECMO therapy
	Emergency treatment of heart failure
	Heart transplant
	Medications for heart failure
Care telemedicine for heart failure, smart medicine, and	Related care, palliative care, and quality of life in patients with heart failure
	Care and patient education in heart failure
	Research on artificial intelligence and heart failure
	Remote and home monitoring of heart failure
	Precision medicine and telemedicine
COVID-19 and heart failure	COVID-19 and heart failure
Risk support factors tools for heart failure and decision	Biomarkers and predictive models of heart failure
	Effect of obesity and lipids on heart failure
	Impact of environmental factors on cardiovascular health
	Changes in the cardiovascular system during aging

MRI: Magnetic resonance imaging; TAVR: Transcatheteraortic valve replacement; ECMO: Extracorporeal membrane oxygenation; COVID-19: corona virusdisease 2019.

While focusing on the main research areas, some research findings with relatively few outcomes should not be dismissed. We calculated the annual IDF for each keyword, indicating that while this keyword may have appeared in previous research, it appeared most frequently in each year to locate those areas that, despite their small number, changed more over time. Table 3 indicates the keywords that have appeared most frequently in their field each year (2007–2021), such as “vericiguat,” “severe acute respiratory syndrome coronavirus 2,” and “atherosclerotic cardiovascular disease” in the last 3 years. The complete list of keywords for 1944–2021 is provided in the Appendix (Table S2).

**Table 3:** Themost frequently used keywords in 2007–2021

Year	Research directions
2021	Vericiguat
2020	Severe acute respiratory syndrome coronavirus 2
2019	Atherosclerotic cardiovascular disease
2018	Cardiac risk factors and prevention
2017	Periodic breathing
2016	Sacubitril/valsartan (LCZ696)
2015	Biological markers
2014	Biological markers
2013	Angioplasty
2012	Heart atria
2011	Angioplasty
2010	Health-care disparities
2009	Angiotensin II receptor blockers
2008	Cardiac myocytes
2007	Menopause

## Discussion

Natural language processing techniques enable the identification of themes and clustering, and detecting clusters across a massive number of heart failure publications. This allows for a macro view of trends in heart failure research. Notably, the research trends in the field of heart failure are gradually changing over time, especially in the last 10 years. The World Health Organization reported a study classifying research findings on cardiovascular disease using a subset of PubMed publications, which required a large expert-based review of a small subset of published articles.^[14]^ This study was updated and more accurate than earlier studies. However, despite the use of advanced automated analytical techniques, experts played a significant role in interpreting and linking concepts to validate the results. Although experts referred to terms on the list of classical ranks in the field while naming current topics and clusters,^[15]^ this approach was used to determine many specific emerging research areas, such as “research on artificial intelligence and heart failure” and “characteristics of heart failure in women.” Therefore, such approaches help provide novel insights. Rather than attempting to categorize all studies, this study captured and identified the most prevalent and evolving themes in the field of heart failure over time by obtaining a comprehensive set of heart failure publications within a certain time frame via authoritative literature databases.

Traditional research on drug therapy, risk factors, arrhythmias, coronary artery disease, metabolic abnormalities, and patient care remains significant in the field. The primary part of this literature is evidence-based treatment guidelines and studies on outcomes and prognosis, both of which must be emphasized.^[16,17]^ This trend was also demonstrated by the growth in evidence-based medicine topics. The rapid growth of health economics, distribution, and use of medical resources, related care for patients with heart failure, palliative care, and quality of life emphasizes patient care, long-term prognosis, and disease care. The importance of preventive medicine is being highlighted by increased research on risk factors, risk scores and classifications, and patient education. Interestingly, research on risk factor-related research areas, such as biomarkers, fat metabolism, glucose metabolism, water–electrolyte balance, and exercise has revealed increasing trends in population, clinical, and basic research areas, implying that heart failure research is more susceptible to cross-domain integration and concatenation.

In the fields of clinical research, artificial intelligence, telemedicine, heart transplantation, stem cells, valve interventional therapy, heart assist devices have advanced rapidly, among other subject areas. These new technologies provide more treatment regimens for patients with severe and end-stage heart failure and support the development of the field of intensive care therapy. They have also provided new impetus to the development of related areas of care, such as palliative care and quality of life for patients with heart failure. These results may also demonstrate the large number of clinical trials being conducted in the field of heart failure to investigate new treatments. Recently, new technologies, devices, and algorithms have been introduced to support this trend.^[18, 19, 20]^ The development of artificial intelligence technology is extremely prominent in the booming trends for new technologies and methods. These fields are concerned with the evolution of diagnosis, treatment models, and basic methods; therefore, these findings will have a profound impact on various fields, including basic and population research.^[21,22]^ The significant achievements of artificial intelligence in recent years have fundamentally altered almost every aspect of heart failure prevention, diagnosis, and management.^[20]^ These findings have prompted an upsurge of new technologies and methods, especially in the fields of diagnostic models, dynamic detection, risk prediction, and prognosis evaluation.^[23, 24, 25]^

Drug toxicity, enzymology, regenerative medicine, and stem cell research are all crucial fields that have rapidly grown in the last 10 years. These related topics are involved in clinical studies, which reflects the rapid development of translational medicine and the rising close relationship between clinical and basic science.^[26,27]^ This is also true for biomarkers, fat metabolism, and genetics-related research. This phenomenon demonstrates the increasing crossover in different directions of heart failure research.

Medical research progress is influenced by factors such as associated needs, technology, funding, and policies.^[5]^ Many countries around the world are currently investing more in clinical research than in basic and population research.^[28,29]^ New methods, drugs, materials, research and development of new devices, and clinical evaluation are all contributing factors to the increase in clinical studies. Previous research occasionally suggests areas that may require additional attention to further contribute to this trend. The available health data and expert opinions are important reference bases for policy designation. For example, heart foundations in the Netherlands^[30]^ and the United Kingdom^[31]^ have consistently prioritized research on heart failure and arrhythmias. Simultaneously, in the context of the global epidemic, studies on the relationship between novel coronaviruses and cardiovascular diseases have increased rapidly, reflecting the situation in which specific medical needs influence the development of academic research.

This study has certain limitations. First, the data included in the study were obtained from Web of Science’s MEDLINE database. However, unlisted articles were not covered in this study. However, we believe that the data from over 270000 articles on heart failure were representative. Second, data from 2022 have not yet been updated; therefore, research hotspots and trends for this year could not be analyzed. Finally, publication outputs are delayed compared with actual research; thus, unpublished papers in ongoing long-cycle research projects cannot be reflected promptly in the results.

This is the largest study on the development trend in the field of heart failure demonstrating that new technologies and personalized and intelligent treatment are receiving increased attention to improve prognoses and quality of life. Medical needs, new technologies, and social support are all driving forces for these developments. Along with expert insights, funding allocation, and policy formulation, these findings will help pave the way for future research.

## Supplementary Information

Figure S1. Flow chart for the data analysis.

Table S1. All of the 107 LDA topics.

Table S2. The complete list of keywords for 1947–2021.

Supplementary information is only available at the official site of the journal at: www.intern-med.com.

## Supplementary Material

Supplementary Material

## References

[1] Institute for Health Metrics and Evaluation (2019). GBD Results Tool.

[2] GBD 2019 Diseases and Injuries Collaborators (2020). Global burden of 369 diseases and injuries in 204 countries and territories, 1990-2019: a systematic analysis for the Global Burden of Disease Study 2019. Lancet.

[3] Roth GA, Mensah GA, Johnson CO, Addolorato G, Ammirati E, Bad-dour LM (2020). Global Burden of Cardiovascular Diseases and Risk Factors, 1990-2019: Update From the GBD 2019 Study. J Am Coll Cardiol.

[4] Baman JR, Ahmad FS (2020). Heart Failure. JAMA.

[5] Gal D, Thijs B, Glänzel W, Sipido KR (2019). Hot topics and trends in cardiovascular research. Eur Heart J.

[6] Heidenreich PA, Bozkurt B, Aguilar D, Allen LA, Byun JJ, Colvin MM (2022). 2022 AHA/ACC/HFSA Guideline for the Management of Heart Failure: Executive Summary: A Report of the American College of Cardiology/American Heart Association Joint Committee on Clinical Practice Guidelines. Circulation.

[7] Synnestvedt MB, Chen C, Holmes JH (2005). CiteSpace II: visualization and knowledge discovery in bibliographic databases. AMIA Annu Symp Proc.

[8] van Eck NJ, Waltman L (2010). Software survey: VOSviewer, a computer program for bibliometric mapping. Scientometrics.

[9] Kozlowski D, Semeshenko V, Molinari A (2021). Latent Dirichlet allocation model for world trade analysis. PLoS One.

[10] Chen ZL (2022). Research and application of clustering algorithm for text big data. Comput Intell Neurosci.

[11] Naeem MZ, Rustam F, Mehmood A (2022). Mui-Zzud-Din, Ashraf I, Choi GS. Classification of movie reviews using term frequency-inverse document frequency and optimized machine learning algorithms. PeerJ Comput Sci.

[12] Heckman GA, Boscart VM, McKelvie RS (2014). Management considerations in the care of elderly heart failure patients in long-term care facilities. Future Cardiol.

[13] Ahmed A (2003). Treatment of chronic heart failure in long-term care facilities: implications of recent heart failure guidelines recommendations. Arch Gerontol Geriatr.

[14] Myers L, Mendis S (2014). Cardiovascular disease research output in WHO priority areas between 2002 and 2011. J Epidemiol Glob Health.

[15] Pries AR, Naoum A, Habazettl H, Dunkel M, Preissner R, Coats CJ (2018). CardioScape mapping the cardiovascular funding landscape in Europe. Eur Heart J.

[16] Djulbegovic B, Guyatt GH (2017). Progress in evidence-based medicine: a quarter century on. Lancet.

[17] Vere J, Gibson B (2019). Evidence-based medicine as science. J Eval Clin Pract.

[18] Vahanian A, Beyersdorf F, Praz F, Milojevic M, Baldus S, Bauersachs J (2022). 2021 ESC/EACTS Guidelines for the management of valvular heart disease. Eur Heart J.

[19] Barreiro-Perez M, Caneiro-Queija B, Puga L, Gonzalez-Ferreiro R, Alarcon R, Parada JA (2021). Imaging in Transcatheter Mitral Valve Replacement: State-of-Art Review. J Clin Med.

[20] Yasmin F, Shah SMI, Naeem A, Shujauddin SM, Jabeen A, Kazmi S (2021). Artificial intelligence in the diagnosis and detection of heart failure: the past, present, and future. Rev Cardiovasc Med.

[21] Howard J (2019). Artificial intelligence: Implications for the future of work. Am J Ind Med.

[22] Chen M, Decary M (2020). Artificial intelligence in healthcare: An essential guide for health leaders. Healthc Manage Forum.

[23] Yang NI, Yeh CH, Tsai TH (2021). Artificial Intelligence-Assisted Identification of Genetic Factors Predisposing High-Risk Individuals to Asymptomatic Heart Failure. Cells.

[24] Shrestha S, Sengupta PP (2018). Imaging heart failure with artificial intelligence: improving the realism of synthetic wisdom. Circ Cardiovasc Imaging.

[25] Ouyang D, He B, Ghorbani A, Yuan N, Ebinger J, Langlotz CP (2020). Video-based AI for beat-to-beat assessment of cardiac function. Nature.

[26] Austin CP (2021). Opportunities and challenges in translational science. Clin Transl Sci.

[27] Pulendran B, Davis MM (2020). The science and medicine of human immunology. Science.

[28] Silvestre J, Abbatematteo JM, Serletti JM, Chang B (2016). National Institutes of Health Funding in Plastic Surgery: A Crisis?. Plast Reconstr Surg.

[29] Head MG, Fitchett JR, Cooke GS, Foster GR, Atun R (2015). Systematic analysis of funding awarded for viral hepatitis-related research to institutions in the United Kingdom, 1997-2010. J Viral Hepat.

[30] Onderzoeksagenda (2018). Hartstichting.

[31] British Heart Foundation (2018). Research Strategy 2015–2020.

