# Supplementary material for: Analysis and prediction of research hotspots and trends in heart failure research
Source: J Transl Int Med. 2024 Jul 27;12(3):263–73. doi: 10.2478/jtim-2023-0117 (PMC11284256; doi:10.2478/jtim-2023-0117)
Supplement: Supplementary file 1 — Supplementary Material [file jtim-2023-0003_sm.pdf]

Analysis and Prediction of Research Hotspots and Trends in Heart Failure Research

Running title: AI-based insight into the wave of heart failure research

This PDF file includes:

Supplemental Figures and Figure Legends

Figure S1

Supplemental Tables and supporting information

Table S1-S2

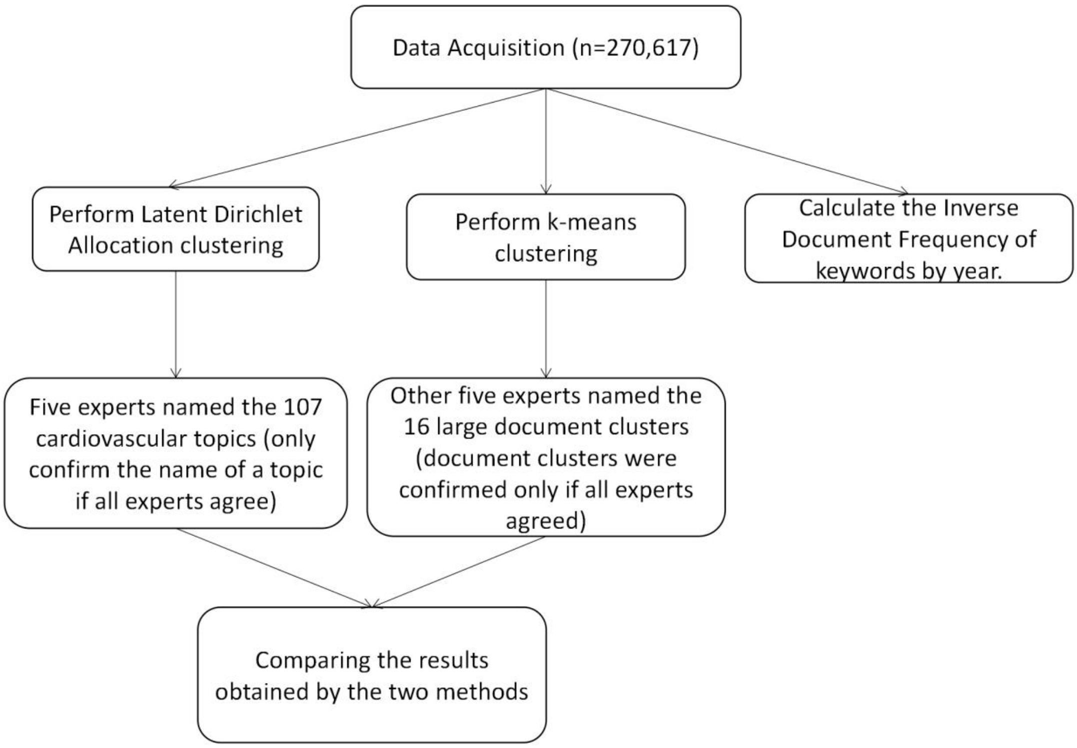

Figures S1. Schematic diagram of the main flow of the study

Table S1. All of the 107 LDA topics

Research Field	LDA Topics
basic	Stem cells, genetics, and gene editing
basic	The role of enzymes in the progression of heart failure
basic	Immune cells and immune regulation
basic	Programmed cell death
basic	Cells and extracellular matrix
basic	Cell regeneration and signaling pathways
basic	Cardiac hypertrophy, fibrosis, and remodeling
basic	Cardiomyocyte ultrastructure, energy metabolism, and signaling
basic	Gene therapy for heart failure
basic	Hemodynamic studies related to heart failure
basic	Function and role of mitochondria in heart failure
basic	Cardiotoxic effects of drugs
basic	Oxygen metabolism and heart failure
basic	Nitric oxide and heart failure
basic	Genetics and single nucleotide polymorphisms
basic	The role of adipose tissue in heart failure
basic	Translational Research
clinical	COVID-19 and heart failure
clinical	ECMO therapy
clinical	The role of NT-proBNP in the diagnosis and treatment of heart failure

clinical	Methods of monitoring disease conditions
clinical	Infectious diseases and heart failure
clinical	Effect of obesity and lipids on heart failure
clinical	Diabetes and heart failure
clinical	Proteins and enzymes
clinical	Amyloidosis and heart failure
clinical	Treatment of mitral valve lesions - surgical and interventional
clinical	Treatment with radioactive iodine
clinical	Pulmonary hypertension and heart failure
clinical	Peritoneal dialysis treatment
clinical	Hypertension - diagnosis, monitoring, and treatment
clinical	Bone marrow and hematopoiesis
clinical	Coronary artery lesions and heart failure
clinical	Acute coronary syndrome and myocardial injury
clinical	Acute myocardial infarction - interventional and pharmacological treatment
clinical	Coronary lesions - diagnosis, treatment, and prognosis
clinical	Treatment of acute heart failure - characteristics, influencing factors, and prognosis
clinical	Parasitic infections and heart failure
clinical	Mechanism and application of antagonist drugs
clinical	Precision medicine and telemedicine
clinical	Dilated cardiomyopathy - etiology, diagnosis, and treatment
clinical	Methodology of clinical trials
clinical	Metabolism of sodium and heart failure
clinical	Endocrine-related diseases (excluding diabetes) and heart failure
clinical	Coagulation, anticoagulation, and atrial fibrillation
clinical	Characteristics of heart failure in women
clinical	Anemia and heart failure
clinical	Potassium metabolism and cardiotonic drugs - effects on patients with heart failure
clinical	Effect of ejection fraction on the treatment and prognosis of patients with heart failure
clinical	Neurological activity and heart failure
clinical	Renin-angiotensin-aldosterone system
clinical	Changes in the cardiovascular system during aging
clinical	Minimally invasive surgery and assistive devices
clinical	Heart failure due to congenital heart disease
clinical	Pericardial disease and heart failure
clinical	Heart failure due to cardiomyopathy - mechanism, diagnosis, and treatment
clinical	Heart failure after myocardial infarction - treatment and prognosis
clinical	Pacing therapy for arrhythmias
clinical	Treatment of the cardiorenal syndrome
clinical	Heart failure and complications in infants and children
clinical	Remote and home monitoring of heart failure
clinical	Diagnosis and differential methods of heart failure
clinical	Intensive care treatment of heart failure
clinical	Effects of heart failure on the kidneys
clinical	Risk score and stratification of heart failure

clinical	Risk factors and predictive models for heart failure
clinical	Related care, palliative care, and quality of life in patients with heart failure
clinical	Care and patient education in heart failure
clinical	Psychosocial care for patients with heart failure
clinical	Water-electrolyte balance and micronutrient supplementation in heart failure patients
clinical	Care and readmission of patients with heart failure
clinical	Emergency treatment of heart failure
clinical	Management and rehabilitation of heart failure disease
clinical	Biomarkers and predictive models of heart failure
clinical	Risk factors and markers of heart failure
clinical	Minimally invasive treatment of heart failure - transcatheter valve replacement
clinical	Evaluation of drug therapy for heart failure
clinical	Heart failure and liver dysfunction
clinical	Heart failure and respiratory sleep disorders
clinical	Heart failure and thyroid function
clinical	Heart failure and congenital defects
clinical	Heart failure and cardiac tumors
clinical	Heart failure and serum proteins
clinical	Minimally invasive treatment for heart failure - TAVI
clinical	Treatment of heart failure - diuretics
clinical	Treatment of heart failure - ventricular assist device
clinical	Medications for heart failure
clinical	Cardiovascular-associated infectious diseases and heart failure
clinical	Heart transplant
clinical	Cardiac resynchronization therapy for heart failure
clinical	3D Echocardiography and 3D Printing
clinical	Vasoactive drugs and the treatment of heart failure
clinical	Endovascular stents
clinical	Hemodynamics and heart failure
clinical	Cardiovascular effects of blood glucose control drugs
clinical	Evidence-based medical research
clinical	Research on artificial intelligence and heart failure
clinical	Cardiovascular effects of malnutrition
clinical	Imaging - ultrasound, and MRI
clinical	The role of exercise in the diagnosis and rehabilitation of heart failure
clinical	Surgical treatment of aortic lesions and complications
population	Public health, socioeconomics, and digital health
population	Impact of environmental factors on cardiovascular health
population	Cardiovascular effects of air pollution
population	Regional distribution and utilization of healthcare related to heart failure
population	Cost and utilization of health care services related to heart failure
population	Health economics of the treatment and care of heart failure

OCT= optical coherence tomography
COPD=chronic obstructive pulmonary disease
MRI=magnetic resonance imaging
CT=computed tomography
ECG=electrocardiogram
CABG=coronary artery bypass graft
ECMO= Extracorporeal Membrane Oxygenation
TAVI=Transcatheter Aortic Valve Implantation

Table S2. The complete list of keywords for 1947–2021

Year	keywords 1	keywords 2	keywords 3
2021	vericiguat	sars-cov-2	ferroptosis
2020	severe acute respiratory syndrome coronavirus 2	coronavirus disease 2019	coronavirus
2019	atherosclerotic cardiovascular disease	cardiovascular outcome trials	transcription
2018	cardiac risk factors and prevention	acute ischemic stroke	cardiac stem cells
2017	periodic breathing	tachycardia, ventricular	family
2016	LCZ696interN-3	marfan syndrome	kidney failure, chronic
2015	biological markers	myocytes	valve surgery
2014	biological markers	waist circumference	heartware
2013	hazard ratio	hazard ratio	new york heart association
2012	heart atria	dronedarone	rhabdomyolysis
2011	angioplasty	angioplasty	dronedarone
2010	healthcare disparities	dronedarone	eisenmenger syndrome
2009	angiotensin ii receptor blockers	angiotensin ii receptor blockers	myocardial revascularization
2008	cardiac myocytes	cardiac myocytes	renin angiotensin system
2007	menopause	schizophrenia	animals
2006	myocyte	pharmacogenomics	regurgitation

2005	biomedical and behavioral research	health care and public health	death and euthanasia
2004	health care and public health	biomedical and behavioral research	empirical approach
2003	non-programmatic	biomedical and behavioral research	professional patient relationship
2002	non-programmatic	biomedical and behavioral research	empirical approach
2001	nasa discipline cardiopulmonary	non-nasa center	prednisone
2000	empirical approach	nasa discipline cardiopulmonary	non-nasa center
1999	nasa discipline cardiopulmonary	non-nasa center	death and euthanasia
1998	non-nasa center	nasa discipline cardiopulmonary	death and euthanasia
1997	studies	non-nasa center	death and euthanasia
1996	age factors	recommendations	population characteristics
1995	developing countries	population dynamics	age factors
1994	professional patient relationship	contraception	death and euthanasia
1993	population characteristics	demographic factors	cardiovascular effects
1992	nasa discipline cardiopulmonary	signs and symptoms	causes of death
1991	developed countries	population dynamics	studies
1990	leukemia	developed countries	contraception
1989	professional patient relationship	death and euthanasia	empirical approach
1988	developed countries	studies	family planning
1987	population dynamics	demographic factors	causes of death
1986	cardiovascular effects	oral contraceptives--side effects	contraceptive methods--side effects
1985	oral contraceptives--side effects	contraceptive methods--side effects	biomedical and behavioral research
1984	oral contraceptives--side effects	contraceptive methods--side effects	cardiovascular effects
1983	oral contraceptives--side effects	contraceptive methods--side effects	family planning
1982	reproduction	biology	signs and symptoms
1981	population dynamics	demographic factors	causes of death
1980	developing countries	health	delivery of health care
1979	developed countries	family planning	demographic factors
1977	reproduction	family planning	biology
1976	endoscopy	studies	diseases
1975	oral contraceptives--side effects	contraceptive methods--side effects	contraception
1974	age factors	research methodology	reproduction

1973	delivery	catheter	reproduction
1972	oral contraceptives--side effects	contraceptive methods--side effects	cardiovascular effects
1970	united states	family planning	surgery
1969	studies	cardiovascular effects	research methodology
1967	oral contraceptives--side effects	contraceptive methods--side effects	neoplasms
1965	drug therapy	toxicologic report	blood gas analysis
1964	surgery, operative	kidney failure, acute	triamterene
1963	pulmonary valve stenosis	diet therapy	diuretics, mercurial
1962	digitalis/therapy	chlorothiazide/related compounds	heart failure, congestive/blood
1961	heart failure, congestive/complications	heart failure, congestive/diagnosis	diuretics/therapy
1960	heart failure, congestive/complications	heart failure, congestive/physiology	heart failure, congestive/diagnosis
1959	acetazolamide/related compounds	heart failure, congestive/metabolism	heart failure, congestive/pathology
1958	acetazolamide/related compounds	congestive heart failure/metabolism	body fluid balance
1957	body fluid balance	congestive heart failure/metabolism	congestive heart failure/in aged
1956	congestive heart failure/in aged	cardiac glycosides/therapeutic use	strophanthin/therapeutic use
1955	congestive heart failure/metabolism in	diuretics/therapeutic use	electrolytes/metabolism
1954	cardiac glycosides/therapeutic use	congestive heart failure/complications	lungs/diseases
1953	water/metabolism	lungs/diseases	congestive heart failure/metabolism in
1952	resins	congestive heart failure/surgery	congestive heart failure/diagnosis
1951	digitalis and preparations	congestive heart failure/pathology	cor pulmonale
1950	digitalis and preparations	diuresis and diuretics	sodium chloride
1949	heart/insufficiency	diuresis and diuretics	venae cavae/surgery
1948	heart/failure	heart/insufficiency	heart/physiology
1947	heart/insufficiency	heart/failure	diuresis and diuretics

Only the top three of the most frequent keywords are displayed.
